# Editorial: Wastewater treatment & resource recovery technologies

**DOI:** 10.3389/fbioe.2023.1150044

**Published:** 2023-04-05

**Authors:** Jiuyang Lin, Jiangjing Li, Yilin Xu, Ming Xie, Shuaifei Zhao, Wenyuan Ye

**Affiliations:** ^1^ Fujian Provincial Engineering Research Center of Rural Waste Recycling Technology, School of Environment and Resources, Fuzhou University, Fuzhou, China; ^2^ Singapore Membrane Technology Centre, Nanyang Environment and Water Research Institute, Nanyang Technological University, Singapore, Singapore; ^3^ Department of Chemical Engineering, University of Bath, Bath, United Kingdom; ^4^ Institute for Frontier Materials, Deakin University, Geelong, VIC, Australia; ^5^ Fujian Provincial Key Laboratory of Soil Environmental Health and Regulation, College of Resources and Environment, Fujian Agriculture and Forestry University, Fuzhou, China

**Keywords:** heavy metal remediation, microalage harvest, fenton “like” process, electroflocculation, dye degradation, wastewater (sewage) treatment (WWT), resource recovery and recycling, nano-zero valent iron (nZVI)

Wastewaters containing organic matters and/or heavy metals are usually generated in various industrial sectors, including textile, pharmaceutical, food and pesticide (Chen et al., 2020; Cai et al., 2021). The discharge of untreated such wastewaters may cause serious health, safety and environmental concerns. Conventional technologies, including advanced oxidation, coagulation, incineration and adsorption mainly focus on the removal/degradation of the existing pollutants (heavy metal or organic compounds), failing to adequately extract resources from the wastewaters (Lin et al., 2021a; Wen et al., 2022). Furthermore, the conventional treatment methods are often costly, time-consuming and/or energy intensive for wastewater management. Under the concept of advanced sustainability, these contaminated wastewaters are considered as important sources of valuable resources (e.g., water, bioproducts, energy, and nutrients) rather than wastes (Jiang et al., 2018; Kamali et al., 2019; Ye et al., 2020a; Lin et al., 2021b). Therefore, it is of critical importance to develop new technologies and materials for effective wastewater management and resource recovery (Ye et al., 2020b).

Technological innovation can significantly improve pollution removal efficiency and reduce secondary contamination. In general, technological innovation in wastewater treatment and resource recovery mainly involves the coupling of different processes for enhanced performance *via* synergistic effects. Xue et al. applied bioinspired calcium carbonate precipitation (MICP) to capsulize heavy metal ions in metallurgical wastewater by the introduction of ureolytic bacteria (*Sporosarcina pasteurii*), yeast extracts and calcium sources, and achieved 100% remediation efficiency for lead (Pb^2+^) and copper (Cu^2+^) ions under optimal conditions. Wang et al. developed a biogeographic technique called enzyme-induced calcium carbonate precipitation (EICP) for heavy metal remediation. Unlike the MICP technique, EICP uses enzymes to specifically catalyze hydrolysis of urea, exhibiting an enhanced remediation efficiency for Pb^2+^ and Cu^2+^ ions in the high-strength heavy metal-containing wastewaters. Furthermore, Alarifi et al. summarized the application of EICP technology in the oil and gas industry, where EICP can effectively solve and prevent sand production Research Topic in oil and gas wells. In addition, innovations are also being made to find more economical and rational strategies for resource recovery from wastewater. Hou et al. integrated alkali-induced flocculation and electrolysis, called salt-bridge electroflocculation (SBEF) with non-sacrificial carbon electrodes for harvesting microalgae. A salt bridge was specifically implemented to connect the two chambers, reducing exogenous contamination, cell oxidative damage and electrode depletion. Such a method showed a high recovery (90.4%) of microalgae with low energy consumption (1.5 Wh/g biomass).

Furthermore, innovations in nano-materials have also been made in parallel to advances in remediation processes to enhance the wastewater remediation efficacy. Fang et al. fabricated a biochar-loaded nano-zerovalent iron composite through homogenous incorporation of nano-zerovalent iron onto chicken manure biochar for remediation of wastewater loaded with heavy metal (i.e., Cr). The biochar-loaded nano-zerovalent iron composite exhibited a considerably high adsorption Cr VI) capacity of 124.1 mg g^-1^ under acidic conditions. Specifically, the removal efficiency of Cr VI) from wastewater reached 98.92% within 72 h. Wu et al. prepared a PEGylated Cu_2_O@SiO_2_/MnO_2_ nanocomposite with an average diameter of 52 nm, *via* a wet chemical route. The nanocomposite can prevent Cu_2_O from oxidation by a dense SiO_2_ shell, imparting stable photo-Fenton-like catalytic activity. Specifically, 92.5% of methylene blue can be degraded in the presence of H_2_O_2_ under visible light for 30 min with a rate constant of 0.086 min^-1^, remarkably outperforming the previously-reported state-of-the-art nano-catalysts.

This Research Topic discusses the latest innovations of remediation technologies and nanomaterials that enable efficient wastewater treatment and resource recovery. In the future, advanced materials and technologies with high cost-effectiveness, ease of expansion and sustainability will provide viable solutions to efficient wastewater treatment and resource recovery. The cost-effective strategy for constructing materials with strong durability/stability in extreme environments and innovations in practical remediation processes are two key research directions that should be focused on. Such advancement will allow wastewater treatment and resource recovery technologies to be reliably applied to a wide range of real wastewaters for minimizing their environmental impacts.
